# Health-related quality of life in women with endometriosis: a systematic review

**DOI:** 10.1186/1757-2215-5-29

**Published:** 2012-10-18

**Authors:** Shuang-zheng Jia, Jin-hua Leng, Jing-hua Shi, Peng-ran Sun, Jing-he Lang

**Affiliations:** 1 Department of Obstetrics and Gynecology, Peking Union Medical College Hospital, Chinese Academy of Medical Science and Peking, Union Medical College, P. R. China

**Keywords:** Endometriosis, Quality of life, Pelvic pain, Endoscopic surgery, Questionnaires

## Abstract

Endometriosis has critical implications for women’s quality of life. However, an overview of the current knowledge of this issue is limited. The objective of this systematic review was to determine the extent of endometriosis and its treatment upon women’s health-related quality of life (HRQoL). PubMed, Embase, PsycoINFO, CINAHL and the Cochrane Clinical Trials were searched up to May 2012, and only studies using standardized instruments to evaluate HRQoL in women with endometriosis were selected. Our electronic searches identified 591 citations, of which 39 studies satisfied the inclusion criteria including nine qualitative studies and 30 treatment-related studies. Findings showed that endometriosis impaired women’s HRQoL. Pain was strongly related to a poor HRQoL, and medical or surgical treatment could partially restore this impairment. No conclusive evidence was available on whether endometriosis imposed an additional impairment in HRQoL per se, apart from the decrease caused by chronic pelvic pain, or on the superiority of various hormonal suppression agents. The impacts of disease extent, duration and fertility status upon HRQoL were inconsistent. In summary, HRQoL was impaired in women with endometriosis, and medical or surgical treatment to alleviate pain could partially restore this impairment.

## Introduction

Endometriosis affects about 10% of women of child-bearing age. Pain symptoms (dysmenorrhea, dyspareunia and dyschezia) may significantly affect their physical, mental and social wellbeing [1]. It is also a major cause of infertility, which in turn causes psychological stress, low self-esteem and depression [2]. Endometriosis is often associated with diagnosis delay and high recurrence rates, leaving women frustrated and catastrophized [3,4]. Moreover, this decreased quality of life often predicts women’s quality-adjusted life-years lost and health care costs [5]. Thus, endometriosis should be analyzed by a bio-psycho-social approach and requires individualized treatment [1,3].

Health-related quality of life (HRQoL) is a multi-dimensional, dynamic concept that encompasses physical, psychological and social aspects associated with a disease or its treatment [6]. Over the last two decades, there has been a growing trend to incorporate assessment of HRQoL into clinical studies and routine clinical management, including that for endometriosis [7]. However, these studies have been limited by small sample sizes, inadequately validated instruments, and heterogeneities between cases and controls. Meanwhile, the impacts of confounders such as age, income, symptom severity, care-seeking behavior and disease extent upon HRQoL in endometriosis are uncertain [4].

Given this background, an updated critical overview of the current knowledge of HRQoL in endometriosis and the effect of treatment is considered timely. In this systematic review, we therefore identified and evaluated studies in which standardized instruments were used to assess this subject.

## Materials and methods

This systematic review was conducted following the MOOSE consensus statement [8]. No ethical approval was needed.

### Data sources

Comprehensive literature searches were performed in Medline/PubMed, Embase, CINAHL, PsycINFO and the Cochrane Clinical Trials database up to May 2012, using the search terms ‘quality of life’, ‘health-related quality of life’, ‘health status measurement’, ‘functional status’ and ‘subjective health status’ in combination with ‘endometriosis’. Additional articles were identified by manually searching references from the retrieved eligible articles. Only peer-reviewed articles published in the English language were included.

### Study selection and data extraction

Studies were selected in a two-stage process. First, the electronic searches were scrutinized and full manuscripts of all citations that were likely to meet the predefined selection criteria were obtained by two reviewers. Second, the reviewers inspected all of the manuscripts to determine whether they met the following criteria: (i) Participants: women with surgical and/or histological diagnosis of endometriosis, with a minimal sample size of 30; (ii) Interventions: standardized instruments/questionnaires to measure HRQoL; (iii) Outcomes: HRQoL measurements by more than one dimension; and (iv) Study design: no study design restriction.

For any duplicate publications, the most complete and relevant versions were included. We extracted data on general study characteristics, patients’ demographics, and questionnaires used systematically onto an extraction sheet.

### Methodological quality assessment

All manuscripts meeting the selection criteria were assessed for their methodological quality, using a 14-item standardized checklist with small modifications as listed in Table 1[9,10]. When a study met the criteria strictly, one point was assigned; if not, the default was 0. A study was considered to be of ‘high quality’ if it scored ≥ 10 points. Studies scoring 7–10 points were rated as ‘moderate quality’, while those scoring < 7 points were deemed of ‘low quality’ [9,10].

**Table 1:** **14-item criteria assessing the methodological quality of included studies**[9,10]

1	Socio-demographic and medical data are clearly described
2	Inclusion and/or exclusion criteria are formulated
3	The process of data collection is described (e.g. Interview or self-report, etc.)
4	The type of treatment is described
5	The results are compared between two groups or more
6	Mean or median and range or standard deviation of time since diagnosis or treatment is given.
7	Participation and response rates for patient groups have to be described and have to be >75%.
8	Information is presented about patient/disease characteristics of respondents and non-respondents or if there is no selective response.
9	A standardized or valid HRQoL questionnaire is used.
10	Results are described not only for HRQoL but also for the physical, psychological and social domains.
11	Key findings are clearly stated.
12	An attempt is made to find a set of determinants with the highest prognostic value.
13	Patient signed an informed consent form before study participation.
14	The degree of selection of the patient sample is described.

### Data synthesis

We identified potential effectors on HRQoL in endometriosis by defining five levels of evidence [9,11] as follows: (i) strong evidence, meaning consistent findings (≥ 75%) in at least two high quality studies; (ii) moderate evidence, meaning consistent findings (≥ 75%) in one high quality study and at least one low quality study; (iii) weak evidence, meaning findings in one high quality study or consistent findings (≥ 75%) in at least three or more low quality studies; (iv) inconclusive evidence, meaning inconsistent findings, or fewer than three low quality studies available; and (v) no evidence with no data presented. We did not attempt to pool data across studies because of the substantial heterogeneities in patient characteristics and choice of HRQoL instruments.

## Results

### Literature identification, study characteristics and quality

**Figure 1:**
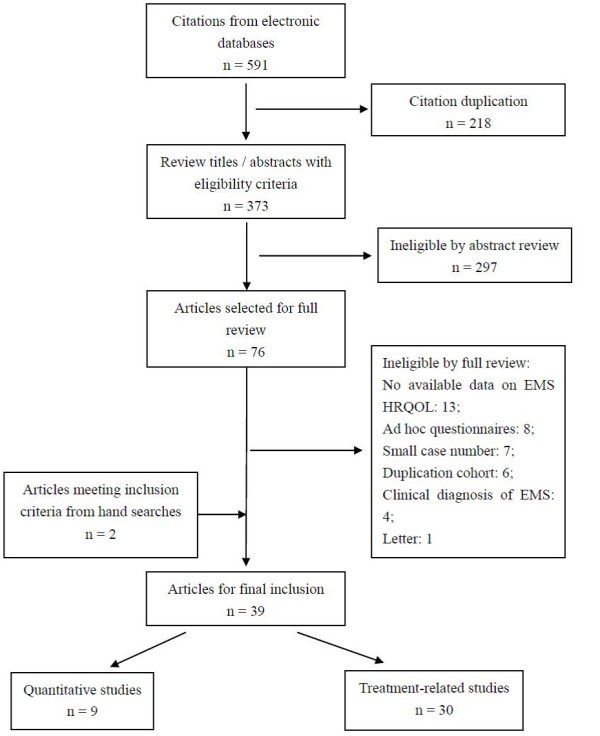
Study selection process for HRQoL in endometriosis.

The flow diagram of literature identification and selection is summarized in Figure 1. A total of 591 citations were identified by electronic searches. After detailed evaluation and inspection of the manuscripts, 39 primary articles met the inclusion criteria. The patient sample size of the included studies ranged from 33 to 1418, with methodological quality scores ranging from 6 to 13. The salient features of each study are provided in Additional file 1and2.

Of these studies, nine aimed to quantify the impact of endometriosis upon HRQoL, with [4,12-15] or without [3,5,16,17] comparison to women without endometriosis. The remaining 30 studies were treatment-related discussing the impact of various interventions upon HRQoL in endometriosis: medical treatment studies [18-31]; surgical treatment studies [32-44]; and complementary therapy studies [45-47]. The extent of endometriosis, demographics, symptoms and endometriosis treatments upon women’s HRQoL are listed in Tables 2, 3, 4.

**Table 2:** HRQoL impairment in women with endometriosis

	**Evidence**	**Reference**
EMS vs. healthy	Strong ↓	[3-5,13,14,16,17]
EMS-related CPP vs. CPP	Inconclusive ↓	[4,12,13]
EMS-related vs. idiopathic infertility	Weak ↓	[15]

Abbreviations: EMS, endometriosis; CPP, chronic pelvic pain; ↓, more impairment.

**Table 3:** Impact of demographics and symptoms on HRQoL

**Evidence**	**Strong**	**Moderate**	**Weak**	**Inconclusive**	**No**	**Reference**
Age				X↓		[3,16]
Income		X-				[3,4]
Education		X-				[3,4]
Employment			X↑			[4]
Marital status		X-				[3,16]
Fertility status				X↓		[3,16,17]
rAFS stage				X↓		[3,4,13,15,16]
Pain intensity	X↓					[3,4,12-14,16]
Duration				X↓		[3,4,16]
Exercise			X↑			[16]

Abbreviations: ↑, positively associated with HRQoL; -, no association with HRQoL; ↓, negatively associated with HRQoL.

**Table 4:** Impact of endometriosis treatment upon HRQoL

**Evidence**	**Strong**	**Moderate**	**Weak**	**Inconclusive**	**Reference**
Medical treatment					
GnRHa	X↑				[18-20,22-27,29]
Add-back vs. GnRHa	X↑				[22,28]
Progestins vs. GnRHa	X↑-				[20,23-25,27,29]
OCs vs. GnRHa				X↑-	[22,26]
Add-back vs. OCs			X↑-		[22]
OCs vs. Progestins			X↑-		[21]
Danazol vs. GnRHa			X↑-		[18]
Surgical treatment					
Conservative	X↑				[26,32-34,36,38,39]
Colorectal resection		X↑			[35,37,40,42-44]
LUNA vs. Conservative			X↑-		[32]
PSN vs. Conservative			X↑		[33]
Complementary treatment					
Dietary			X↑		[26]
Acupuncture			X↑		[45]
CHM			X-		[46]
PMR			X↑		[47]

Abbreviations: ↑, positively associated with HRQoL; -, no association with HRQoL; ↓, negatively associated with HRQoL; ↑-, similar effective in HRQoL improvement; OC, oral contraceptive; LUNA, laparoscopic uterosacral nerve ablation; PSN, presacral neurectomy; PMR, progressive muscular relaxation; CHM, Chinese herb medicine.

### HRQoL instruments and measures for endometriosis

A total of nine instruments for measurement of HRQoL in endometriosis were identified. Generic HRQoL questionnaires - used to capture a broad range of physical, mental and social health variables - were used in all but four studies [28,42,43,46]. The SF-36 was used in more than half of the studies. The Endometriosis Health Profile-30 (EHP-30) is the only validated disease-specific HRQoL scale for use in endometriosis [48,49]. Seven studies explored this instrument [24,25,28,42,43,46] and its brief version [17], the Endometriosis Health Profile-5, to assess HRQoL. A detailed analysis of these instruments is outside the scope of this review.

### HRQoL impairment in women with endometriosis

Endometriosis impaired women’s HRQoL compared with healthy controls [3-5,13,14,16,17]. In 2008, a case–control study in Brazil reported that women with endometriosis and chronic pelvic pain (CPP, n = 93) showed impairment of all domains of the SF-36 (except for role-related emotional functioning) and higher levels of perceived stress than 82 healthy volunteers [14]. Two other studies from Brazil [3,16] also found worse HRQoL scores and higher depression and anxiety rates in endometriosis. It was also reported that endometriosis was associated with greater losses in work productivity [3,4,13,17] and increased sexual dysfunction [13]. Moreover, decreased HRQoL was the most important predictor of health-care cost [5].

However, in comparison to women with similar CPP symptoms, whether endometriosis had more impairment upon HRQoL was inconclusive [4,12,13]. According to the Global Study of Women’s Health, women with endometriosis (n = 745) reported more HRQoL impairment and work productivity loss than those with similar symptoms (n = 587) - especially for the physical component [4]. However, inconsistent findings were reported in the other two studies with small sample sizes [12,13]. Women with CPP attributed to endometriosis or not showed similar scores for HRQoL, anxiety, depression [12] and sexual satisfaction [13].

Infertile women secondary to endometriosis reported higher levels of perceived stress and a lower HRQoL than did women with idiopathic infertility, according to the only available high-quality study from Germany, with no significant difference in social support [15].

### Impact of demographics and symptoms on HRQoL

Women of advanced age were reported to suffer more depressive symptoms than did younger women [3]. However, no such correlation was found by another study [16]. Employed women were significantly less likely to be impacted by endometriosis [4], as were those who took regular exercise [16]. Marital status [3,16], income and education level [3,4] were not associated with HRQoL impairment. However, two of the three studies enrolled women mainly from low-income socioeconomic status groups [3,16].

Pain was strongly related to a poor HRQoL. Women with severe pain were more likely to report higher levels of HRQoL impairment, depression, anxiety, sexual dysfunction, and more work productivity loss [3,4,12-14], although Marques et al. denied this correlation [16]. With respect to infertility, no consistent conclusion could be made [3,16,17]. Infertility significantly impaired the physical and mental status in endometriosis [17]. However, no correlation between psychiatric symptoms and fertility status was observed in one Brazilian study [3], and Marques et al. even reported that women with children scored lower than women without children in the functional capacity dimension, according to the SF-36 [16].

The impacts of rAFS stage [3,4,13,15,16] and disease duration [3,4,16] upon HRQoL were inconsistent. Advanced rAFS stages were reported to negatively influence work productivity and HRQoL [4,15]. In contrast, Marques et al. reported that women with advanced stages of endometriosis scored better on mental health and emotional role limitation domains [16]. However, most studies revealed no correlation between psychiatric symptoms and rAFS stages [3,13]. No correlation between psychiatric symptoms and diagnosis delay was found in two Brazilian studies [3,16], although a multicenter study revealed that diagnostic delay worsened the HRQoL [4].

### Impact of treatment upon HRQoL

#### Medical treatment

Hormonal suppression was strongly associated with HRQoL improvement in women with endometriosis, regardless of the use of gonadotropin releasing hormone agonist (GnRHa) alone or with hormonal add-back therapy, oral contraceptives (OCs) or progestins. However, it should be noted that most data before and after treatment were derived from specific arms of randomized controlled trials (RCTs) with the exception of one prospective cohort study [31].

Six months of GnRHa treatment improved women’s HRQoL even at one year after treatment, according to the SF-36 questionnaire [24-27,29], as well as work-related indexes [18,20]. However, contradictory results were observed with regard to social support [18,20,24,25], sexual function [18,20,24,25] and the psychological general well-being index (PGWBI) [18,23]. A clinically relevant increase in anxiety/depression score was observed during the 6-month GnRHa treatment [20]. Three months of GnRHa treatment was also associated with HRQoL improvement [47], and the pseudomenopausal symptoms it induced caused less deterioration of HRQoL than that caused by surgically induced menopause [31]. Considering 12 months of treatment, satisfaction with pain control was reported during and at 6 months post-treatment [22]. Notably, an increase in pain and a decrease in HRQoL were reported by women during one month of GnRHa therapy [19].

GnRHa plus add-back therapy improved HRQoL in women with recurrent [22] or intractable endometriosis-related pain [28]. Moreover, when compared with GnRHa alone, add-back hormonal therapy was better at improving the HRQoL at both 3 and 12 months of treatment and at 6 months post-treatment [22]. When GnRHa was prescribed for 18 months, significant and similar HRQoL improvement and pain control from baseline to 18 months were observed in both immediate and delayed add-back therapy groups, but the former was associated with better self-image and emotions after a 12-month discontinuation, according to the EHP-30 [28].

Progestins were positively associated with HRQoL in women with endometriosis [20,21,23-25,27,29,30]. Subcutaneous injections of depomedroxyprogesterone acetate (104 mg/0.65 mL) improved all pre-specified scales of the EHP-30 and SF-36 from baseline to the end of the 6-month treatment and even at the 18-month follow-up [24,25]. Similar results were reported for dienogest in women with symptomatic endometriosis [27,29]. Cyproterone acetate therapy also improved the HRQoL, psychiatric profile, and sexual function [21], as well as for medroxyprogesterone acetate therapy [20]. However, the high dropout rate (n = 12/25) in the later study limited the authors from drawing a firm conclusion [20]. Postoperative levonorgestrel intrauterine system (LNG-IUS) for 12 months improved both physical and mental health for women with moderate to severe pain related to endometriosis [30], although inconsistent finding was reported, possibly due to low sensitivity of the PGWBI questionnaire applied [23]. Moreover, six months of therapy with progestins seemed equivalent to that of GnRHa in improving HRQoL and reducing pain, according to six RCTs [20,23-25,27,29]. This effect persisted for as long as 12 months after discontinuation [27,29].

Therapy with OCs also improved the HRQoL in women with endometriosis [21,22,26]. Six months of therapy with OCs produced significant pain control and improvements of all SF-36 domains during treatment [21,26], as well as in psycho-emotional status and sexual function (n = 39) [21] at 12 months after discontinuation (n = 38) [26]. However, Zupi et al. revealed that 12 months of therapy with OCs failed to improve the HRQoL in women with recurrent pelvic pain (n = 43), with the symptoms relapsing after discontinuation [22].

Six months of danazol therapy improved the PGWBI scores significantly from baseline, with no effect on the quality of work or sexual function scores [18].

With regard to different hormonal agents for treating endometriosis, no consistent superiority was observed. GnRHa without add-back therapy produced better pain relief and HRQoL improvement than did OCs in women with pain relapse after conservative surgery [22]. In contrast, postoperative hormonal suppression with either GnRHa or OCs for prophylaxis against pain recurrence showed similar improvement [26]. When considering OCs versus GnRHa plus add-back therapy, 12 months of add-back therapy was reported to produce better pain control, physical function and vitality in patients with pain relapse after conservative surgery [22]. Continuous therapy with OCs was reported to be as effective as progestins therapy in improving HRQoL, psycho-emotional status and sexual function [21].

#### Surgical treatment

Conservative surgery improved HRQoL in endometriosis [26,32-34,36,38,39]. A RCT conducted by Abbott et al. demonstrated that laparoscopic excision of the endometriotic tissues was more effective than placebo treatment for reducing pain and improving HRQoL and sexual activity with a 12-month follow-up, albeit with a 30% placebo response rate [34]. This improvement even persisted for up to 5 years (n = 176) [36]. Similar findings were also reported by Roman et al., especially for adolescent women, with a mean of 37.82 months follow-up [38]. Three other studies (n = 260) with different objectives also demonstrated the positive effects of conservative surgery with up to 24 months of follow-up, although this was not the primary aims of these studies [26,32,33]. With respect to deep infiltrating endometriosis, laparoscopic management was also associated with significant HRQoL improvement [39].

Laparoscopic colorectal resection was strongly related to HRQoL improvement for treating colorectal endometriosis, according to six studies including a total of 446 women [35,37,40,42-44]. Laparoscopic colorectal resection improved all domains of the SF-36 [37,40], and this improvement could persist for up to 55 months [37]. Similar findings were also reported by Meuleman and colleagues using a multidisciplinary laparoscopic approach, giving a high fertility rate (n = 29/61) and gynecological pain relief [42,43]. Moreover, laparoscopic approach offered a higher pregnancy rate than open surgery with similar improvements in symptoms and in HRQoL [35]. However, no correlation between the presence of positive margins and HRQoL amelioration was observed [44].

There was weak evidence that adjunct neurectomies, including laparoscopic uterosacral nerve ablation (LUNA) and presacral neurectomy (PSN), were more effective than simple laparoscopy [32,33]. In 2003, an opened RCT conducted by Vercellini et al. reported that LUNA did not add more HRQoL and sexual function improvement in women with endometriosis and predominantly midline dysmenorrhea, compared with laparoscopic conservative surgery alone [32]. With respect to PSN, better improvements in all domains of the SF-36 were reported at the expense of chronic constipation and/or urinary urgency rate [33].

#### Complementary treatments

Weak evidence existed for the efficacy of complementary treatments such as dietary therapy, acupuncture, progressive muscular relaxation (PMR) and Chinese herbal medicine (CHM) upon HRQoL in endometriosis. Acupuncture was reported to improve all domains of the SF-36 (except for physical role limitation) in women with symptomatic endometriosis [45]. For CHM therapy, the only included RCT failed to reveal any additional benefit [46]. Dietary supplementation for 6 months after conservative laparoscopy showed a better HRQoL improvement than did surgery alone [26]. Recently, a RCT reported that PMR training was more effective in improving anxiety, depression and HRQoL in endometriosis women under GnRHa therapy [47].

## Discussion

To assess endometriosis from the woman’s point of view and to address its associated emotional, sexual and social problems are the primary goals of endometriosis management [13]. In this systematic review, we comprehensively evaluated the impact of endometriosis and its treatment upon women’s HRQoL.

It is not surprising to find that women with endometriosis reported significant impairments in HRQoL, since pelvic pain intensity was negatively associated with HRQoL. This finding was also observed in women with other benign gynecologic conditions, according to a systematic review conducted by Jones et al. [50]. CPP, the main and most wildly studies symptoms of endometriosis, often bothers women with endometriosis for years [4], frustrates both patient and clinician [51], and is associated with work productivity loss and significant physical and social debility [5].

However, the current evidence does not allow us to conclude endometriosis imposed more impairment upon HRQoL than women with similar CPP symptoms. Indeed, CPP is not a disease but a description of a clinical condition. Women with CPP often present some degrees of pain hypersensitivity [52]. And finding in rat model suggests that peripheral and central sensitization is a common sense in endometriosis and other pain syndromes such as painful bladder or irritable bowel syndrome [53]. Moreover, women with endometriosis have a higher rate of other comorbid pain syndromes and may be prone to depression, anxiety and chronic fatigue [53]. Thus, to consider endometriosis-related CPP in the context of chronic pain, and to understand commonalities across different forms of chronic pain might provide an alternative approach to improve HRQoL of these patients [12].

According to our analysis, different treatment hormonal agents showed similar efficacy in pain control and in HRQoL improvement, but with different side effects and cost profiles. OCs and progestins, with good safety profile, low cost and tolerability, appear to be the better choices [20,24-27,29]. When these agents fail, a 12-month treatment with GnRHa is the only recommendation approved by the US FDA [54]. Add-back hormonal therapy should be started immediately [28]. Conservative surgery could enhance women’s fertility and temporally restore HRQoL [1,54]. Adjuvant therapies like CHM and dietary modification that inhibit oxidative stress, angiogenesis and inflammation observed with endometriosis need more study. However, it should be noted that the efficacy is short-term and recurrence is common in both medical and surgical modalities [32,36,38]. Thus, a multi-disciplinary strategy involving a pain clinic and counseling is recommended [1], but unfortunately no comparatively study was available on their impact upon HRQoL.

Currently, there is no gold standard to assess HRQoL in women with endometriosis. We identified a total of nine instruments to measure HRQoL in endometriosis with different conceptual frameworks, scales, response formats and scoring systems. However, their psychometric properties and internal consistency were not well established in endometriosis [50]. Generic instruments such as the SF-36 are useful for comparing between different disorders; however, they correlate poorly with pain intensity and are compromised by the use of medications in endometriosis [16,36]. Moreover, issues important and unique to endometriosis, such as infertility, might not be addressed by generic questionnaires [48]. The EHP-30, based on open-ended exploratory interviews with patients, is currently the only validated questionnaire for endometriosis [48]. Thus, the EHP-30 is recommended in HRQoL research on endometriosis, and a combination with generic instruments is needed when comparing between different disorders.

Our systematic review has several limitations. First, the high degree of heterogeneity with respect to study design, patients’ demographics, disease severity, measures and data presentation hampers interpretation and synthesis of the included studies. Further, no gold standards exist to assess the study quality related to HRQoL [55]. The 14 quality criteria we used have good reliability to discover predictors of HRQoL impairment [10]. Moreover, the majority of the studies we reviewed did not correct for possible confounders and determinants with the highest prognostic value and no sample calculations were described, although, to some extent, these confounder problems offset in the data from RCTs. Besides, unpublished studies were not identified, which could lead to publication bias.

In summary, endometriosis impairs women’s HRQoL and this impairment can be at least partly and temporally counteracted repaired with hormonal therapy and conservative surgery. However, whether endometriosis itself imposes additional HRQoL impairment in women with CPP, or on the superiority of various hormonal suppression agents is not conclusive. Prospective study designs, appropriate adjustment for confounding factors, diverse patient populations, and the use of validated and disease-specific instruments such as EHP-30 would greatly enhance our understanding of HRQoL in endometriosis.

## Abbreviations

HRQoL: Health-related quality of life; SF-36: The Short Form – 36; EHP-30: The Endometriosis Health Profile – 30; CPP: Chronic pelvic pain; GnRHa: Gonadotropin releasing hormone agonist; OCs: Oral contraceptives; PGWBI: The psychological general well-being index; RCT: Randomized controlled trial; LUNA: Laparoscopic uterosacral nerve ablation; PSN: Presacral neurectomy; PMR: Progressive muscular relaxation; CHM: Chinese herbal medicine.

## Competing interests

None of the authors have a conflict of interest.

## Authors' contributions

S-zJ contributed substantially to conception and design analysis, electronic searches, data extraction, drafted the article, and revised and approved the final version to be published. J-hL contributed substantially to conception and design and approved the final version to be published. J-hS performed the study selection and data extraction, revised the article, and approved the final version to be published. P-rS contributed to the data extraction and approved the final version to be published.J-hL contributed substantially to conception and design and approved the final version to be published. All authors read and approved the final manuscript.

## Supplementary Material

Additional file 1 Studies assessing HRQoL in women with endometriosis, with or without comparisons. Click here for file

Additional file 2 Studies assessing interventions on HRQoL in women with endometriosis. Click here for file
