# Supplementary material for: Health-related quality of life in women with endometriosis: a systematic review
Source: J Ovarian Res. 2012 Oct 18;5:29. doi: 10.1186/1757-2215-5-29 (PMC3507705; doi:10.1186/1757-2215-5-29)
Supplement: Additional file 1 — Studies assessing HRQoL in women with endometriosis, with or without comparisons. [file 1757-2215-5-29-S1.docx]

**Additional file 1** Studies assessing HRQoL in women with endometriosis, with or without comparisons

| Reference | Population (No.) | Measurement | Associated factors | Main HRQoL findings | Quality |
| --- | --- | --- | --- | --- | --- |
| Cross-sectional studies | | | | | |
| [16] | EM-CPP (57) | SF-36 | Stage, exercise, children | Poor HRQoL in EMS. | 10 |
| [3] | EMS (104) | WHQOL-BREF | Pain, treatment duration | Poor HRQoL in EMS. | 11 |
| [17] | EMS (193) | SF-12, EHP-5 | NR | Poor HRQoL in EMS. | 6 |
| [12] | CPP (57) | WHQOL-BREF | Pain | Pain reduced HRQoL. | 11 |
| [13] | CPP and healthy (134) | WHQOL-BREF | Pain | Pain reduced HRQoL. | 9 |
| [4] | CPP and healthy (1418) | SF-36 | Pain, employment, diagnostic delay, stage | Poorer HRQoL in EMS. | 13 |
| [5] | EMS (909) | EQ-5D | NR | Poor HRQoL in EMS. | 10 |
| Case-control study | | | | | |
| [14] | EM-CPP and healthy (175) | SF-36 | Pain, stress | Poorer HRQoL in EMS. | 11 |
| [15] | Infertility (69) | SF-36 | Stage | Poorer HRQoL in EMS. | 11 |

Abbreviations: EMS, endometriosis; EM-CPP, endometriosis-related chronic pelvic pain; SF-36, the Short Form-36; WHOQOL-BREF, the short (26-item) version of the World Health Organization quality of life instrument; EHP-5, the Endometriosis Health Profile-5; EQ-5D, the European Quality of life Scale; NR, not reported.
