# Supplementary material for: Health-related quality of life in women with endometriosis: a systematic review
Source: J Ovarian Res. 2012 Oct 18;5:29. doi: 10.1186/1757-2215-5-29 (PMC3507705; doi:10.1186/1757-2215-5-29)
Supplement: Additional file 2 — Studies assessing interventions on HRQoL in women with endometriosis. [file 1757-2215-5-29-S2.docx]

**Additional file 2** Studies assessing interventions on HRQoL in women with endometriosis.

| Reference | Patients (No.) | Intervention (duration) | Measurement | Follow-up | Main HRQoL benefits | Quality |
| --- | --- | --- | --- | --- | --- | --- |
| Randomized Control Trials | | | | | | |
| [18] | EMS (169) | GnRHa vs. Danazol (6 M) | PGWBI | 1 M | Similar improvement. | 7 |
| [19] | EM-CPP (120) | GnRHa vs. Placebo (1 M) | SF-36 | No | Decreased HRQoL. | 10 |
| [20] | EM-CPP (48) | GnRHa vs. MPA (6 M) | NHP | 6 M | Similar improvement. | 10 |
| [21] | Recurrent pain (90) | CPA vs. OC (6 M) | SF-36 | No | Similar improvement, favored CPA. | 13 |
| [32] | EMS, dysmenorrhea (180) | Lap. vs. LUNA | SF-36 | 12 M | Similar improvement. | 13 |
| [33] | EMS, dysmenorrhea (141) | Lap. vs. PSN | SF-36 | 24 M | Better with PSN. | 13 |
| [34] | EMS (39) | Lap. vs. Placebo | SF-12, EQ-5D | 12 M | Significant improvement, with 30% placebo effect. | 12 |
| [22] | Recurrent pain (133) | GnRHa vs. OC vs. Add-back (12 M) | SF-36 | 6 M | Better with add-back. | 13 |
| [23] | EMS, pain (82) | GnRHa vs. LNG-IUS (6 M) | PGWBI | No | Similar improvement, not significant. | 9 |
| [24] | Persistent pain (300) | GnRHa vs. DMPA-SC (6 M ) | SF-36, EHP-30 | 12 M | Similar improvement. | 11 |
| [25] | Persistent pain (274) | GnRHa vs. DMPA-SC (6 M) | SF-36, EHP-30 | 12 M | Similar improvement. | 10 |
| [26] | rAFS III-IV EMS (222) | Lap. plus placebo vs. GnRHa vs. OC vs. dietary therapy (6 M) | SF-36 | 12 M | Better with postoperative hormonal or dietary. | 10 |
| [27] | EMS, pain (271) | Dienogest vs. GnRHa (6 M) | SF-36 | No | Similar improvement, favored dienogest. | 11 |
| [28] | EM-CPP (38) | Immediate vs. delayed add-back (18 M) | EHP-30 | 12 M | Better with immediate add-back. | 11 |
| [29] | EMS, pain (252) | Dienogest vs. GnRHa (6 M) | SF-36 | No | Similar improvement, favored dienogest. | 13 |
| [35] | Colorectal EMS (52) | Lap. vs. laparotomic colorectal resection | SF-36 | 6 – 36 M | Similar improvement. | 12 |
| [45] | EMS, pain (101) | Acupuncture vs. Placebo (5 weeks) | SF-36 | 6 M | Better with acupuncture. | 10 |
| [46] | EM-CPP (33) | CHM vs. Placebo (4 M) | EHP-30 | No | Similar improvement, favored CHM. | 10 |
| [30] | EM-CPP (55) | Lap. plus LNG-IUS vs. Lap. (12 M) | SF-36 | No | Better with LNG-IUS. | 12 |
| [47] | EMS, pain (100) | GnRHa plus muscle relaxation vs. GnRHa (3 M) | SF-36 | No | Better with muscle relaxation. | 9 |
| Prospective Cohort Studies | | | | | | |
| [36] | EM-CPP (176) | Lap. | SF-12, EQ-5D | 2-5 years | Improved significantly. | 10 |
| [37] | Colorectal EMS (93) | Lap. colorectal resection | SF-36 | 2 – 55 M | Improved significantly. | 8 |
| [31] | EMS (61) | GnRHa-induced vs. surgical menopause | MRS-II | 6 M | Better with pseudomenopause. | 10 |
| [38] | EMS (163) | Lap. | EQ-5D | 35 – 41 M | Improved significantly. | 10 |
| [39] | DIE (100) | Lap. | SF-36 | 6 M | Improved significantly. | 12 |
| [40] | Colorectal EMS (151) | Lap. colorectal resection | SF-36 | 12 M | Improved significantly. | 9 |
| Retrospective Cohort Studies | | | | | | |
| [41] | RV-DIE (44) | Surgery | EQ-5D, | 2-22 M | Better with hysterectomy and rectal resection. | 7 |
| [42] | Colorectal EMS (56) | Lap. colorectal resection | EHP-30 | 6 −76 M | Improved significantly. | 7 |
| [43] | Colorectal EMS (47) | Lap. colorectal resection | EHP-30 | 16 – 40 M | Improved significantly. | 8 |
| [44] | Colorectal EMS (47) | Lap. colorectal resection | SF-36 | 6 – 35 M | Improved significantly. | 11 |

Abbreviations: EMS, endometriosis; PGWBI, the Psychological General Well-Being Index; M, month; EM-CPP, endometriosis-related chronic pelvic pain; SF-36, the Short Form-36; MPA, medroxyprogesterone acetate; NHP, the Nottingham Health Profile; CPA, cyproterone acetate; OC, oral contraceptive; Lap, laparoscopy; LUNA, laparoscopic uterosacral nerve ablation; PSN, presacral neurectomy; EQ-5D, the European Quality of life Scale; LNG-IUS, levonorgestrel intrauterine system; DMPA-SC, subcutaneous depot depomedroxyprogesterone acetate; EHP-30, the Endometriosis Health Profile-30; MRS-II: the Menopause-Rating Scale.
